# A preoperative nomogram for predicting 2-year postoperative recurrence after percutaneous transforaminal endoscopic decompression in degenerative lumbar spinal stenosis

**DOI:** 10.3389/fradi.2026.1821920

**Published:** 2026-05-04

**Authors:** Xinyi Luo, Yiwen Wang, Lele Xue, Qi Liu, Yue Yang, Qin Zhang, Shiwu Yin

**Affiliations:** 1 Anhui Medical University, Hefei, China; 2 Department of Interventional Vascular Pain, The Second People’s Hospital of Hefei, Hefei, Anhui, China

**Keywords:** clinical prediction model, degenerative lumbar spinal stenosis, nomogram, percutaneous transforaminal endoscopic decompression, postoperative recurrence, risk stratification

## Abstract

**Background and objectives:**

Postoperative recurrence after percutaneous transforaminal endoscopic decompression (PTED) for degenerative lumbar spinal stenosis (DLSS) remains a clinically relevant challenge, complicating preoperative counseling and long-term management. Reliable tools for predicting individual 2-year recurrence risk using routinely available preoperative data are currently lacking. This study aimed to develop and internally validate a practical preoperative nomogram for individualized recurrence risk prediction after PTED.

**Methods:**

We conducted a retrospective cohort study including 206 patients with DLSS who underwent single-level PTED between August 2021 and August 2023. Preoperative clinical and imaging variables were extracted to construct a multivariable logistic regression model. Candidate predictors were prespecified based on clinical relevance and routine availability. Model performance was evaluated in terms of discrimination, calibration, and clinical utility. Internal validation was performed using 1000 bootstrap resamples and leave-one-out cross-validation (LOOCV).

**Results:**

During the 2-year follow-up period, 29 patients (14.08%) experienced postoperative recurrence. The final nomogram incorporated five preoperative predictors: body mass index, diabetes mellitus, lumbosacral transitional vertebrae, number of levels with senior grade facet degeneration, and paraspinal skeletal muscle index. The model showed good discrimination, with an area under the receiver operating characteristic curve (AUC) of 0.845 (95% CI, 0.778–0.912). Bootstrap validation showed a mean AUC of 0.842 (95% CI, 0.772–0.912), and LOOCV yielded an AUC of 0.797 (95% CI, 0.716–0.878). Calibration was satisfactory, and decision curve analysis demonstrated net clinical benefit across a wide range of threshold probabilities.

**Conclusions:**

We developed a clinically interpretable preoperative nomogram that reliably predicts 2-year postoperative recurrence after PTED in patients with DLSS. By integrating routinely assessed clinical and imaging factors, this tool may facilitate individualized risk stratification, support informed preoperative counseling, and guide risk-adapted perioperative management. External validation in independent cohorts is warranted.

## Introduction

Degenerative lumbar spinal stenosis (DLSS) is a leading cause of pain, neurogenic claudication, and functional decline in aging populations, with prevalence rising sharply with age (1–4). Although clinically recognizable, DLSS is heterogeneous in its structural patterns and clinical progression. Stenosis can vary across anatomical compartments, and patients with similar imaging findings often experience markedly different symptom severity and postoperative outcomes. This variability suggests that uniform surgical approaches and follow-up protocols may fail to address long-term risks in clinical practice (1, 3, 4).

Minimally invasive endoscopic decompression, particularly percutaneous transforaminal endoscopic decompression (PTED), has gained adoption for its ability to minimize surgical trauma while effectively addressing neural compression, especially in the lateral recess and foramen (5–9). Systematic reviews indicate that endoscopic techniques yield symptom improvement comparable to microscopic or minimally invasive decompression, with potential advantages in reduced complications and faster recovery for suitable patients (5–8). Nevertheless, procedural durability remains a significant challenge, as postoperative recurrence and the need for reintervention in a subset of patients can diminish the long-term benefits of a less-invasive approach, leading to additional risk, cost, and functional decline (10–13).

Recurrence following PTED is unlikely to stem from a single factor but rather emerges from the interplay of multiple domains: systemic vulnerability, lumbosacral anatomy, cumulative degenerative load, and musculoskeletal reserve (12–15). “Host factors” are increasingly recognized in this process. Obesity, linked to poorer outcomes after lumbar stenosis surgery in large registries, may exert influence through elevated mechanical stress and systemic inflammation (16). Similarly, diabetes mellitus has been associated with worse outcomes in various spinal surgeries, likely due to microvascular compromise, impaired healing, and altered pain perception (17, 18). These systemic factors are at least partly modifiable, highlighting a practical connection between preoperative risk assessment and perioperative optimization.

Paraspinal muscle reserve is another critical but often overlooked determinant of postoperative resilience. Quantitative imaging measures of paraspinal muscle mass and quality correlate with disability and recovery outcomes across degenerative lumbar conditions (19, 20). Diminished muscle reserve may weaken dynamic spinal stability, hinder functional recovery after decompression, and reflect broader physiological frailty not captured by routine demographics. Together, these observations support a model where postoperative recurrence is influenced by both local structural pathology and patient-specific resilience—factors that are ascertainable preoperatively and thus suitable for personalized risk estimation.

Despite this growing understanding of recurrence as a multifactorial process, clinicians still lack practical tools to estimate individualized recurrence risk after PTED using routinely available preoperative information. Many studies focus on isolated predictors or single-domain imaging features, and existing models may face barriers to routine clinical adoption, including limited interpretability, incomplete calibration assessment, or uncertain utility across clinically relevant decision thresholds (10, 11, 21). Effective prediction tools for patient counseling and risk-adjusted management require transparent development, and should be evaluated not only for discriminative ability but also for calibration and net clinical benefit (21, 22). To address this gap, we developed and internally validated a pragmatic clinicoradiological nomogram to predict postoperative recurrence after PTED for DLSS. The model utilizes routinely available preoperative clinical and imaging variables, with the goal of enabling individualized risk stratification and supporting risk-adapted perioperative optimization and postoperative surveillance planning.

## Materials and methods

### Study design and participants

This retrospective cohort study enrolled consecutive patients who received single-level percutaneous transforaminal endoscopic decompression (PTED) at our center from August 2021 to August 2023. The primary surgical indication was symptomatic unilateral foraminal or lateral recess stenosis associated with lumbar disc herniation. Patients were eligible for inclusion if they met the following criteria: they were aged 18 years or older; presented with unilateral radicular symptoms that correlated with MRI findings of stenosis at the corresponding level; had endured symptoms for a minimum of 3 months without sufficient relief from conservative therapy; and were undergoing their first PTED procedure. Importantly, only patients who demonstrated significant clinical improvement immediately after surgery were considered to be at risk for subsequent recurrence and were included in the follow-up analysis. Patients without initial postoperative improvement were excluded because they were not considered at risk for true postoperative recurrence, thereby avoiding misclassification of early treatment failure as recurrence. Key exclusion criteria comprised the presence of other spinal pathologies such as tumor, infection, or acute fracture; spondylolisthesis exceeding Meyerding grade I; symptomatic stenosis affecting multiple lumbar levels; and any history of prior lumbar spine surgery. To ensure a consistent endpoint definition, patients without available 24-month outcome information were excluded from the primary analysis. Patients without available 24-month outcome information were excluded from the primary analysis. This study was performed in line with the principles of the Declaration of Helsinki and received formal approval from our Institutional Ethics Committee (Approval No. 2025-Research-095).

### Surgical procedure and follow-up

Patients were placed in a lateral decubitus position for the procedure. The surgery employed a standard transforaminal approach, with all key steps performed under continuous fluoroscopic (x-ray) guidance. Following standard skin preparation and draping, the surgical area was locally anesthetized using lidocaine, infiltrating along the planned needle trajectory and around the facet joint. A spinal needle was first advanced to reach the target superior articular process under x-ray control. Once its position was confirmed as correct, a guidewire was inserted. Sequential dilators were then passed over the guidewire to create a pathway, culminating in the placement of a working cannula. The endoscopic system and surgical reamers were introduced through this cannula. Under combined anteroposterior and lateral fluoroscopic views, the final position of the cannula tip was adjusted. On the AP view, it was placed near the posterior midline, and on the lateral view, it was situated within the spinal canal, posterior to the vertebral body line. The herniated disc material was first identified endoscopically. Decompression was then carried out by meticulously removing the extruded nucleus pulposus piece by piece using pituitary forceps. Any hypertrophic ligamentum flavum or degenerative annular tissue contributing to nerve root compression was carefully ablated and contoured with a radiofrequency probe. The endpoint was visual confirmation of a freely mobile nerve root and restored dural pulsation. For cases involving foraminal stenosis, the neural foramen was enlarged (foraminoplasty) with a reamer as needed. In lateral recess stenosis, partial resection of the relevant bony structures—including hypertrophic portions of the facet joint—was performed using reamers and endoscopic osteotomes to adequately expand the recess. After achieving satisfactory decompression, final hemostasis was obtained. The disc annulus and any remaining nucleus material were contoured with the radiofrequency probe. Low-temperature plasma coagulation was used as an adjunct for additional hemostasis when necessary. Finally, the endoscope and working cannula were withdrawn, and the small skin incision was closed with a sterile dressing. All procedures were performed by experienced surgeons following a standardized PTED protocol at our institution. Patients were scheduled for postoperative follow-up for up to 24 months. Follow-up assessments were conducted through outpatient visits, supplemented by electronic medical record review, and telephone interviews when in-person visits were not feasible. Patients without available outcome information at 24 months were excluded from the primary analysis.

### Outcome definition: postoperative recurrence

Postoperative recurrence was defined according to a set of specific clinical and radiographic criteria. First, it was required that initial postoperative imaging confirmed successful decompression at the operated level, showing either improvement or resolution of the original disc herniation and relief of neural compromise, which was accompanied by a corresponding period of meaningful symptom relief. Following this, patients were required to have experienced a sustained period of clinical success—defined as being either symptom-free or having only minimal, non-disabling symptoms—for a minimum of three months after surgery (23). Recurrence was then considered only if, after this interval, there was a clear worsening or return of symptoms, such as radicular pain or neurogenic claudication, that represented a significant decline from the postoperative baseline. Finally, any such recurrent symptoms had to be corroborated by new imaging findings. Specifically, MRI or CT evidence demonstrating pathology—such as re-herniation or re-stenosis—at the same spinal segment that was originally operated on was required to confirm the recurrence was at the index level.

### Clinical and imaging variables

Preoperative clinical data were gathered from a thorough review of patient medical records. This included standard demographic details such as age, sex, and body mass index (BMI). Clinical history documented the duration of symptoms and relevant habits like smoking. Comorbid conditions, notably diabetes mellitus and hypertension, were recorded. Baseline pain severity was quantified using the Visual Analogue Scale (VAS), and the operative time for the procedure was also noted. Imaging variables were obtained from standard preoperative radiographs, computed tomography (CT), and magnetic resonance imaging (MRI) studies. Assessments included the grading of lumbar spinal stenosis and the characteristics of the disc herniation. The degree of intervertebral disc degeneration and facet joint osteoarthritis were graded. Other evaluated features comprised Modic changes in the vertebral endplates, the calculated paraspinal skeletal muscle index (SMI), and the presence of anatomical variants like lumbosacral transitional vertebrae (LSTV). Quantitative measures such as the Disc Height Index (DHI) were also derived from the imaging studies.

### Imaging assessment and definitions

We reviewed all preoperative imaging studies—including lumbar radiographs, CT, and MRI scans—to evaluate the spinal canal anatomy and document key degenerative changes. The severity of stenosis was classified on T2-weighted MRI sequences according to an established morphology-based system. This system categorizes the constriction of the dural sac and nerve roots within specific anatomical compartments: the central canal, lateral recess, and neural foramen. For analytical purposes, these grades were simplified into a binary classification. Central canal stenosis was categorized as low-grade (grades 0–2) or high-grade (grades 3–4). Similarly, lateral recess stenosis was defined as low-grade (grades 0–1) or high-grade (grade 2), and foraminal stenosis as low-grade (grades 0–1) or high-grade (grades 2–3). Degeneration of the intervertebral discs was graded on sagittal T2-weighted MRI using the Pfirrmann classification. Concurrently, facet joint osteoarthritis was evaluated on axial CT images based on the Weishaupt grading scale. We also recorded the presence and type of Modic changes as markers of vertebral endplate degeneration. Furthermore, we documented specific features of the disc herniation itself from the MRI reports. These included the spinal level of the herniation, its morphology (such as protrusion vs. extrusion), and its precise location and direction relative to the neural structures. Imaging assessments were performed by trained investigators who were blinded to postoperative outcomes.

### Degenerative burden measures

To assess the overall burden of spinal degeneration, we calculated a simple count of severely affected lumbar segments. We considered a disc severely degenerated if it scored a Pfirrmann grade of IV or V. Similarly, a facet joint was classified as having severe degeneration if it received a Weishaupt grade of II or III. These counts—representing the total number of segments with severe disc degeneration and the total number with severe facet joint degeneration—were then included in our statistical model as straightforward numerical variables. The counts of severe facet degeneration and severe disc degeneration were calculated across all lumbar segments, including the index surgical level.

## Paraspinal skeletal muscle index (SMI)

Paraspinal muscle reserve was evaluated using the skeletal muscle index (SMI). This index was calculated as the total cross-sectional area of the paraspinal muscles (in mm^2^) measured on lumbar axial MRI slices, divided by the square of the patient's height (in m^2^) (1):

$$\mathrm{SMI}=\frac{\mathrm{TCSA}\;(mm^{2})}{\mathrm{heigh}t^{2}\;(m^{2})}$$

To quantify paraspinal muscle cross-sectional area, axial T2-weighted MRI slices at the mid-L3/4 disc level were analyzed. These images were processed using the ImageJ software (version 1.54) (24). The bilateral multifidus and erector spinae muscles were manually outlined on each side by tracing their contours to define the regions of interest. From these tracings, the cross-sectional areas of the left and right paraspinal muscles were quantified, and the bilateral total cross-sectional area (TCSA) was calculated as the sum of both sides for each patient (see Supplementary Figure S1). The mid-L3/4 level was selected because it is commonly used as a representative landmark for paraspinal muscle assessment and has been shown to correlate with overall lumbar muscle reserve (25). SMI measurements were performed by multiple trained investigators who were blinded to postoperative outcomes.

### Lumbosacral transitional vertebrae (LSTV)

The assessment for lumbosacral transitional vertebrae (LSTV) was performed by reviewing available preoperative imaging, primarily standard lumbar radiographs and supplemented by cross-sectional CT or MRI studies when necessary for confirmation, following established diagnostic criteria (26). When an LSTV was identified, its specific subtype—classified as either sacralization or lumbarization—was documented. This subclassification was collected to allow for potential exploratory analysis of outcomes between these anatomical variants at a later stage.

### Disc height index (DHI)

Disc height index (DHI) was measured on lateral lumbar radiographs using the following formula:

$$\mathrm{DHI}=\frac{(\mathrm{Ha}+\mathrm{Hp})}{(\mathrm{Ds}+\mathrm{Di})}\;\times \;100\text{\%}$$

where Ha and Hp represent anterior and posterior disc heights, and Ds and Di represent superior and inferior disc depths, respectively.

### Statistical analysis and model development

Statistical analysis was conducted using SPSS version 27.0 (IBM) and R version 4.5.1. The distribution of continuous variables was evaluated with the Shapiro–Wilk test. Based on their distribution, data are presented as either mean ± standard deviation or median with interquartile range (IQR). For comparisons between groups, continuous variables with a normal distribution were analyzed using the independent-samples *t*-test, while non-normally distributed variables were compared using the Mann–Whitney *U* test. Categorical variables are summarized as frequencies and percentages, with comparisons performed using the Chi-square test or Fisher's exact test, as appropriate. A two-sided *P* value < 0.05 was considered statistically significant.

### Predictor identification and multivariable modeling

Recurrence (coded as 1 for yes and 0 for no) was the primary outcome. Candidate predictors were prespecified *a priori* based on clinical relevance and routine preoperative availability, with univariable comparisons used for descriptive purposes and to inform (but not determine) model specification. We fitted a multivariable logistic regression model and assessed multicollinearity using variance inflation factors (VIF); all retained predictors had VIF <5. The final model included five predictors, and the regression coefficients from the adjusted model were translated into a points-based nomogram using the rms package in R.

### Penalized regression and sensitivity analysis

To evaluate the robustness of the variable selection process, we also performed a penalized regression analysis using the least absolute shrinkage and selection operator (LASSO) method. The optimal regularization parameter (*λ*) was determined through k-fold cross-validation, applying the widely used one-standard-error rule. This criterion selects the most parsimonious model whose performance is within one standard error of the model with the minimum cross-validation error. Predictors with regression coefficients shrunk to zero were excluded from the LASSO model. The LASSO analysis was used as a supplementary method to assess the stability of predictor selection and to support the specification of the final multivariable model.

### Model performance, internal validation, calibration, and clinical utility

Model discrimination was assessed using the area under the receiver operating characteristic curve (AUC), and the 95% confidence interval for the AUC was calculated using the DeLong method. Model calibration was evaluated using calibration plots and the Hosmer–Lemeshow goodness-of-fit test; overall calibration accuracy was additionally quantified by the mean absolute error (MAE) of the optimism-corrected calibration curve. Given the limited number of recurrence events, the model was developed using the full dataset rather than randomly splitting the cohort into separate training and testing sets, as data partitioning could further reduce the effective sample size and compromise model stability. To mitigate overfitting and obtain more robust performance estimates, internal validation was performed using 1,000 bootstrap resamples and LOOCV. Finally, decision curve analysis (DCA) was conducted to evaluate the net benefit of the model across a range of threshold probabilities and was compared with the default strategies of treating all patients vs. treating none.

## Results

### Recurrence rate and baseline characteristics

The analysis included 206 patients with adequate 24-month outcome ascertainment. The cohort comprised 132 men and 74 women, with an average age of 56.11 years (standard deviation, 10.76; range 41–84). During follow-up, 29 patients (14.08%) experienced recurrence, whereas 177 did not. Comparisons of baseline clinical characteristics between the recurrence and non-recurrence groups showed that most variables were similar between the two groups (Table 1). However, patients in the recurrence group had a higher body mass index (BMI) and a higher prevalence of diabetes mellitus than those in the non-recurrence group.

**Table 1:** Comparison of baseline clinical and imaging characteristics between the non-recurrence and recurrence groups.

Variable	Non-recurrence group (*n* = 177)	Recurrence group (*n* = 29)	Statistic (t/*χ*^2^)	*P value*
Sex, *n* (%)			0.030	0.862
Female	64 (36.16)	10 (34.48)		
male	113 (63.84)	19 (65.52)		
Age (years), mean ± SD	56.12 ± 10.98	56.07 ± 9.44	0.023	0.982
BMI (kg/m^2^), mean ± SD	24.03 ± 2.83	25.99 ± 3.97	−3.247	0.001**
Symptom duration (months), mean ± SD	34.02 ± 50.32	45.02 ± 55.14	−1.076	0.283
Operative time (min), mean ± SD	74.38 ± 18.75	70.00 ± 19.32	1.161	0.247
Education level, *n* (%)			−0.252	0.801
Primary school or below	49 (27.68)	5 (17.24)		
Secondary school	90 (50.85)	20 (68.97)		
College/University or above	38 (21.47)	4 (13.79)		
Smoking status, *n* (%)			0.134	0.714
No	122 (68.93)	19 (65.52)		
Yes	55 (31.07)	10 (34.48)		
Alcohol, *n* (%)			0.037	0.847
No	149 (84.18)	24 (82.76)		
Yes	28 (15.82)	5 (17.24)		
Hypertension, *n* (%)			1.354	0.245
No	134 (75.71)	19 (65.52)		
Yes	43 (24.29)	10 (34.48)		
Diabetes mellitus, *n* (%)			6.895	0.024*
No	166 (93.79)	23 (79.31)		
Yes	11 (6.21)	6 (20.69)		
Preoperative VAS score, mean ± SD	5.55 ± 0.65	5.52 ± 0.78	0.230	0.818
Operated level, *n* (%)			0.399	0.819
L3/4	2 (1.13)	0 (0.00)		
L4/5	92 (51.98)	16 (55.17)		
L5/S1	83 (46.89)	13 (44.83)		
Type of lumbar disc herniation, *n* (%)			1.029	0.310
Protrusion	161 (90.96)	28 (96.55)		
Extrusion	16 (9.04)	1 (3.45)		
Lumbar spinal stenosis classification				
Central canal stenosis (MRI grade), *n* (%)			0.354	0.552
Low-grade (grades 0–2)	102 (57.63)	15 (51.72)		
High-grade (grades 3–4)	75 (42.37)	14 (48.28)		
Lateral recess stenosis (MRI grade), *n* (%)			0.305	0.581
Low-grade (grades 0–1)	94 (53.11)	17 (58.62)		
High-grade (grade 2)	83 (46.89)	12 (41.38)		
Foraminal stenosis (MRI grade), *n* (%)			0.000	0.997
Low-grade (grades 0–1)	55 (31.07)	9 (31.03)		
High-grade (grades 2–3)	122 (68.93)	20 (68.97)		
Modic changes, *n* (%)			0.502	0.918
None	107 (60.45)	18 (62.07)		
Type I	2 (1.13)	0 (0.00)		
Type II	68 (37.85)	11 (37.93)		
LSTV, *n* (%)			13.811	<0.001**
Absent	146 (82.49)	15 (51.72)		
Sacralization of L5	24 (13.56)	12 (41.38)		
Lumbarization of S1	7 (3.95)	2 (6.90)		
Number of segments with high-grade disc degeneration (Pfirrmann grades 4–5), mean ± SD	2.07 ± 0.96	1.86 ± 1.19	1.062	0.290
Number of segments with high-grade facet joint degeneration (Weishaupt grades 2–3), mean ± SD	1.15 ± 1.12	1.72 ± 1.03	−2.584	0.010*
SMI(mm^2^/m^2^), mean ± SD	1,580.94 ± 266.18	1,425.85 ± 266.61	2.908	0.004**
DHI(%), mean ± SD	21.21 ± 4.18	22.90 ± 4.79	−1.981	0.049*

Data are presented as mean ± SD or *n* (%).

BMI, body mass index; VAS, visual analog scale for low back pain.

SMI, skeletal muscle index of the lumbar paraspinal muscles; DHI, disc height index; LSTV, lumbosacral transitional vertebra. Central canal stenosis was graded on MRI as low-grade (0–2) or high-grade (3–4). Lateral recess stenosis was graded as low-grade (0–1) or high-grade (2). Foraminal stenosis was graded as low-grade (0–1) or high-grade (2–3). High-grade disc degeneration was defined as Pfirrmann grades 4–5, and high-grade facet joint degeneration as Weishaupt grades 2–3.

* *P* < 0.05, **P < 0.01.

### Preoperative imaging characteristics

Preoperative imaging findings were also compared between the two groups (Table 1). Patients in the recurrence group had a higher prevalence of lumbosacral transitional vertebrae (LSTV), a greater number of segments with high-grade facet joint degeneration, a higher disc height index (DHI), and a lower lumbar paraspinal skeletal muscle index (SMI) than those in the non-recurrence group. No significant between-group differences were observed for type of lumbar disc herniation, MRI-based grades of central canal stenosis, lateral recess stenosis, or foraminal stenosis, presence of Modic changes, or number of segments with high-grade disc degeneration.

### Predictor identification and model robustness assessment

Based on the preoperative clinical and imaging characteristics, we fitted a multivariable logistic regression model to identify factors independently associated with recurrence within 2 years after PTED. Recurrence was entered as the binary outcome variable. Multicollinearity diagnostics showed no concerning collinearity among the predictors included in the final model, with all variance inflation factors below 5 (Table 2).

**Table 2:** Collinearity analysis of predictive factors.

Predictor	Tolerance	VIF
BMI	0.906	1.104
SMI	0.928	1.077
Diabetes	0.986	1.014
LSTV	0.977	1.024
Number of high-grade small joint degeneration	0.989	1.011

The final multivariable model identified five independent predictors of recurrence (Table 3). Higher body mass index (BMI) was associated with higher odds of recurrence (OR, 1.301 per 1 kg/m^2^ increase; 95% CI, 1.080–1.528; *P* = 0.001). Diabetes mellitus was also associated with increased recurrence risk (OR, 3.670; 95% CI, 1.076–12.514; *P* = 0.038). Among the imaging-related variables, the presence of lumbosacral transitional vertebra (LSTV) was associated with higher odds of recurrence (OR, 4.167; 95% CI, 1.550–10.212; *P* = 0.004), and a greater number of levels with high-grade small joint degeneration was likewise associated with increased recurrence risk (OR, 1.671; 95% CI, 1.062–2.388; *P* = 0.024). In contrast, a higher lumbar paraspinal skeletal muscle index (SMI) was associated with lower odds of recurrence (OR, 0.996; 95% CI, 0.994–0.998; *P* < 0.001).

**Table 3:** Multivariable logistic regression analysis of recurrence after PTED in patients with DLSS.

Predictor	*β*	SD	OR	95% CI	*P* value
BMI	0.263	0.085	1.301	1.08–1.528	0.001
SMI	−0.004	0.001	0.996	0.994–0.998	<0.001
Diabetes	1.300	0.626	3.670	1.076–12.514	0.038
LSTV	1.381	0.481	4.167	1.550–10.212	0.004
Number of high-grade small joint degeneration	0.465	0.207	1.671	1.062–2.388	0.024

BMI, body mass index; SMI, skeletal muscle index of the lumbar paraspinal muscles; LSTV, lumbosacral transitional vertebra; OR, odds ratio; CI, confidence interval.

**Figure 1:**
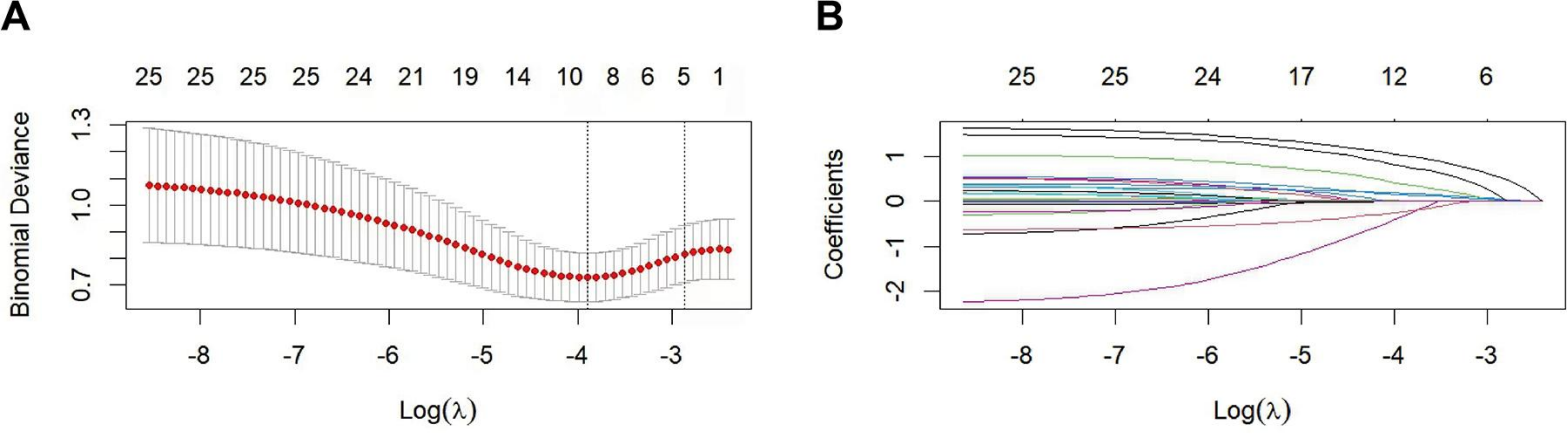
LASSO-based feature selection for postoperative recurrence prediction after PTED. **(A)** Cross-validation curve of binomial deviance plotted against log(λ) in LASSO logistic regression. Red dots indicate the mean cross-validated deviance, and grey bars represent ±1 standard error. The numbers shown above the plot indicate the number of predictors with non-zero coefficients at each value of λ. The vertical dotted lines indicate the optimal *λ* values selected by the minimum deviance criterion and the 1-standard-error rule. **(B)** Coefficient profiles of candidate predictors as a function of log(λ), showing coefficient shrinkage and variable selection during the LASSO procedure. LASSO, least absolute shrinkage and selection operator; PTED, percutaneous transforaminal endoscopic decompression.

To further assess the stability of predictor selection, least absolute shrinkage and selection operator (LASSO) logistic regression with cross-validation was performed (Figures 1A,B). The LASSO analysis supported the specification of the final five-predictor model.

### Nomogram construction for individualized recurrence risk prediction

**Figure 2:**
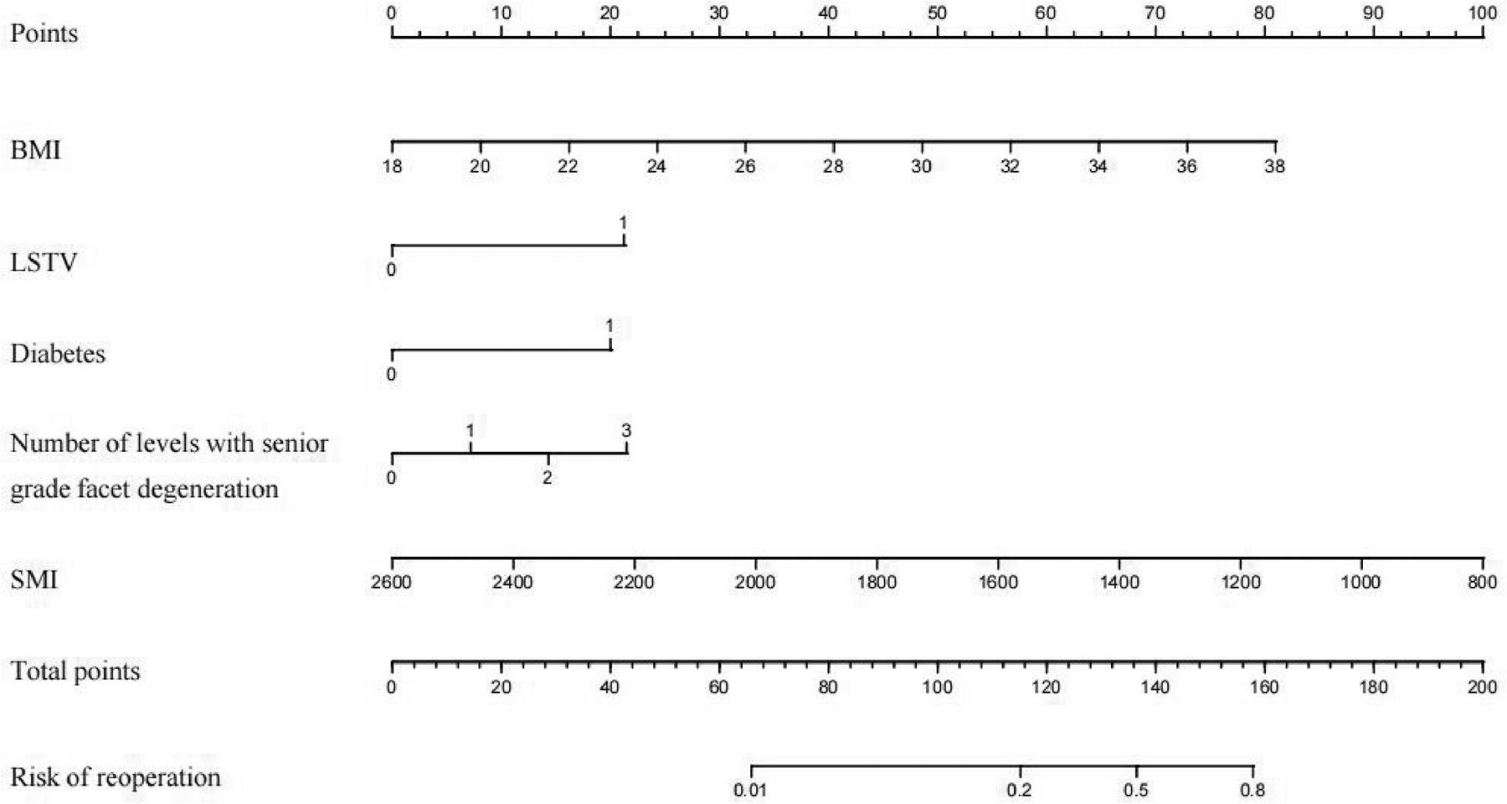
Nomogram for predicting postoperative recurrence after PTED in patients with DLSS. The nomogram includes BMI, LSTV, diabetes, number of levels with senior grade facet degeneration, and SMI. The points assigned to each predictor are summed to obtain the total points, which are then converted into the estimated risk of postoperative recurrence.

Based on the five independent predictors identified in the final model (Table 2), we constructed a nomogram to estimate an individual patient's risk of reoperation within 2 years after PTED (Figure 2). The nomogram was developed using the rms package in R. Each predictor—BMI, LSTV, diabetes, number of levels with senior grade facet degeneration, and SMI—was assigned a corresponding point value according to its contribution to the model. The total points, calculated by summing the score for each predictor, were then mapped to the estimated probability of reoperation. In the nomogram, diabetes and LSTV contributed relatively higher point values, followed by BMI and number of levels with senior grade facet degeneration, whereas a higher SMI corresponded to fewer points.

### Model performance, calibration, and decision curve analysis

**Figure 3:**
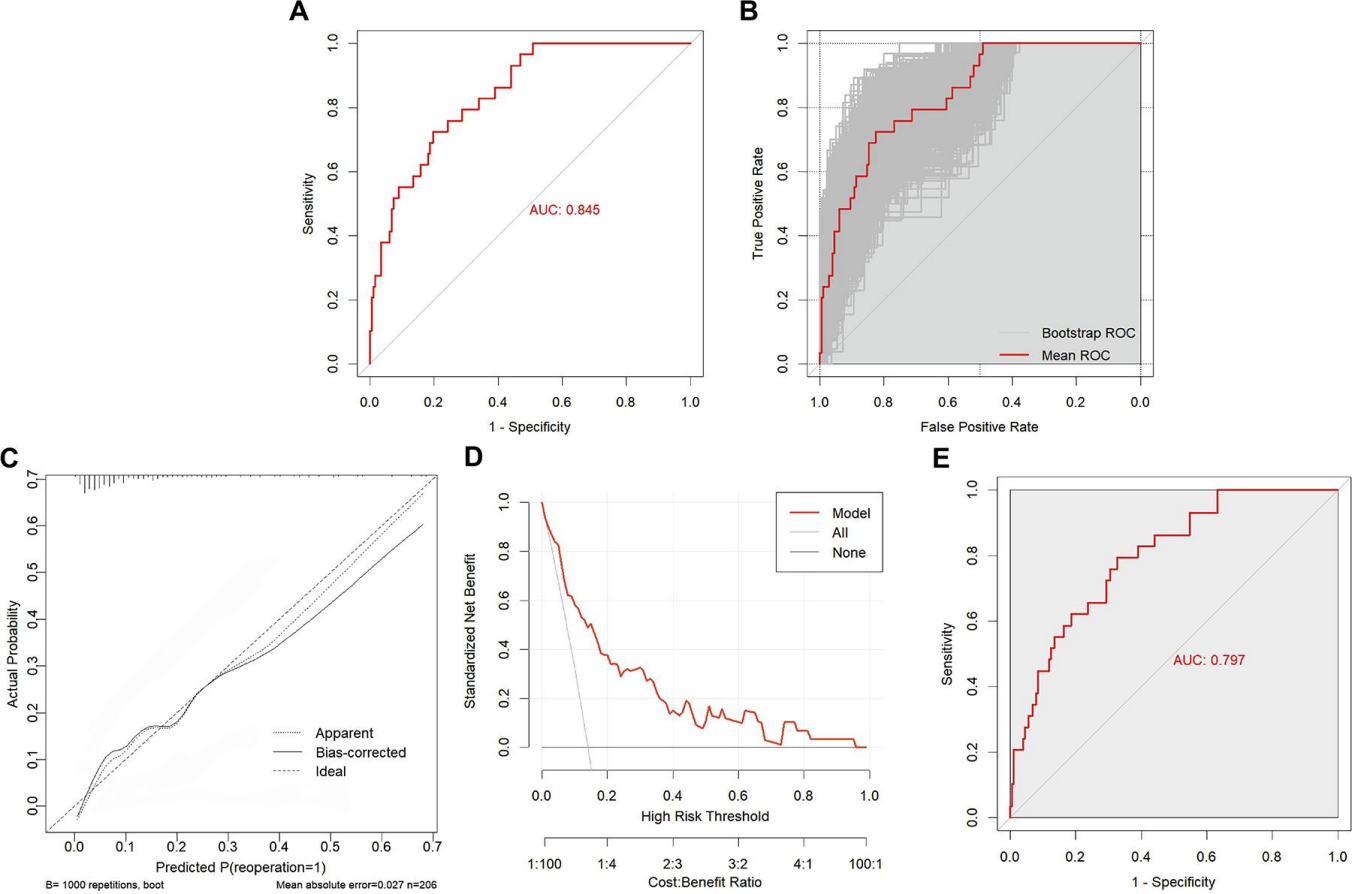
Performance evaluation, internal validation, calibration, and clinical utility of the nomogram for predicting postoperative recurrence after PTED in patients with DLSS. **(A)** ROC curve of the five-predictor nomogram in the full cohort (AUC, 0.845; 95% CI, 0.778–0.912). **(B)** Bootstrap ROC analysis based on 1,000 resamples (mean AUC, 0.842; 95% CI, 0.772–0.912). **(C)** Bootstrap-corrected calibration plot showing agreement between predicted and observed probabilities (*B* = 1,000; *n* = 206). **(D)** Decision curve analysis showing the net benefit of the nomogram relative to the treat-all and treat-none strategies. **(E)** LOOCV ROC curve of the nomogram (AUC, 0.797; 95% CI, 0.716–0.878).

After constructing the nomogram incorporating five predictors, we evaluated its performance in terms of discrimination, calibration, and clinical utility (Figure 3). The area under the receiver operating characteristic curve (AUC) was 0.845 (95% CI, 0.778–0.912) (Figure 3A). At the maximum Youden index of 0.526, the sensitivity was 0.724 and the specificity was 0.802. Internal validation using 1,000 bootstrap resamples yielded a mean AUC of 0.842 (95% CI, 0.772–0.912) (Figure 3B). The bootstrap-corrected calibration curve showed agreement between the predicted and observed 2-year recurrence probabilities (Figure 3C). The Hosmer–Lemeshow goodness-of-fit test was not statistically significant (*χ*^2^ = 10.186, *P* = 0.252). Decision curve analysis showed a higher net benefit of the nomogram than the treat-all and treat-none strategies over a threshold probability range of 0.01–0.96 (Figure 3D). Leave-one-out cross-validation yielded an AUC of 0.797 (95% CI, 0.716–0.878) (Figure 3E).

## Discussion

The durability of decompression for degenerative lumbar spinal stenosis (DLSS) continues to be challenged by postoperative recurrence and reintervention, even with the growing adoption of endoscopic and other minimally invasive techniques (27–29). In this study, we developed a clinical prediction tool that integrates routinely available preoperative data into an interpretable nomogram, providing an individualized estimate for symptomatic, same-level structural recurrence following percutaneous transforaminal endoscopic decompression (PTED). To strengthen the model's relevance for clinical practice, our approach extended beyond conventional metrics. We incorporated penalized regression to assess the stability of predictor selection, performed internal validation using bootstrap resampling and leave-one-out cross-validation, and evaluated clinical utility through decision curve analysis (21).

The predictors retained in the final model jointly delineate a coherent, multifactorial framework for recurrence risk that can be organized into four interrelated domains: systemic metabolic burden (BMI and diabetes), lumbosacral anatomic context (LSTV), posterior-element degenerative load (severe facet degeneration burden), and paraspinal musculoskeletal reserve (SMI). Prior evidence links these domains to longer-term outcomes and the risk of reintervention after lumbar spine surgery, underscoring their relevance to procedural durability (16, 30–32). Although LSTV subtypes were recorded in this study, they were not analyzed separately because the limited sample size and number of recurrence events would have resulted in insufficient statistical power for reliable subtype-specific inference. Future studies with larger cohorts are needed to determine whether sacralization and lumbarization confer different patterns of biomechanical stress and recurrence risk.

A deliberate and important aspect of our study was the definition of the primary outcome. We focused on “index-level symptomatic structural recurrence”—characterized by a clear symptom relapse after a period of postoperative improvement, supported by imaging confirmation of recurrent pathology at the originally operated level—rather than using reoperation as the endpoint. This definition captures the core biological event of interest, aligning with key components (same level, symptom return, imaging proof) commonly used in definitions of recurrent disc pathology (33). While studies vary in their required symptom-free interval (from shorter periods in endoscopic literature to the traditional 6-month benchmark) (10, 34), we employed a pragmatic 3-month window. This aims to minimize misclassifying early incomplete recovery as true recurrence while remaining feasible within standard clinical follow-up. In contrast, the decision to reoperate is influenced by numerous factors such as symptom severity, patient preference, surgical judgment, and healthcare access, which can separate the biological event of recurrence from the subsequent surgical intervention (35–37). By anchoring the model to structural recurrence rather than reoperation, we sought to improve its interpretability and potential applicability across clinical settings with different thresholds for revision surgery.

Regarding our analytical approach, we used LASSO penalized regression as a supplementary method to assess the stability of predictor selection, rather than as the sole determinant of model specification. LASSO is designed for prediction optimization in the context of correlated variables, where it may retain one representative predictor from a correlated group (38). In line with current guidelines that advocate for transparent model reporting beyond mere performance statistics (39), we considered the LASSO results alongside the final multivariable model. In the revised analysis, the final nomogram was constructed based on a five-predictor model including BMI, diabetes, LSTV, number of levels with senior grade facet degeneration, and SMI. The LASSO results were broadly consistent with this final specification, supporting both the robustness and interpretability of the selected predictors. This interpretability is vital for patient communication and for connecting risk estimates to potentially modifiable factors. Accordingly, we used decision curve analysis to evaluate whether applying the nomogram for clinical decisions would provide a net benefit across a range of realistic risk thresholds (21). In addition, internal validation using bootstrap resampling and leave-one-out cross-validation further supported the stability of model performance.

From a practical standpoint, this nomogram could serve several purposes: (1) guiding preoperative optimization for higher-risk patients (e.g., encouraging weight management, glycemic control, or prehabilitation), (2) personalizing postoperative surveillance plans with a lower threshold for re-imaging in high-risk individuals, and (3) facilitating risk enrichment in future studies evaluating recurrence-prevention strategies. The role of prehabilitation before lumbar surgery is an active area of investigation with evolving evidence (40, 41), underscoring the importance of pairing risk prediction with evaluations of targeted interventions.

Our study has several important limitations. First, its retrospective, single-center design may affect the generalizability of the findings. Second, the number of recurrence events was relatively small, and although bootstrap resampling and leave-one-out cross-validation were performed, some degree of overfitting or optimism may still remain. Third, we modeled recurrence as a binary outcome within a 2-year window rather than analyzing time-to-event. Fourth, although imaging measurements, including SMI, were performed by multiple trained investigators, we did not formally assess inter-rater reliability. Fifth, the degenerative burden measures included the index surgical level, which may partly capture local disease severity at the treated segment and thus introduce bias. We did not perform a separate analysis excluding the index level. Therefore, external validation in larger, multicenter prospective cohorts with standardized outcome definitions and reliability testing is planned as an important next step to assess the generalizability of the model before broader clinical application.

## Conclusion

We developed a practical, points-based nomogram based on routinely available clinical and imaging variables to estimate the risk of same-level symptomatic recurrence after PTED in patients with DLSS. The model demonstrated good discriminative performance, satisfactory calibration, and favorable clinical utility, with internal validation performed using bootstrap resampling and leave-one-out cross-validation. By enabling individualized risk stratification, this tool may support preoperative patient counseling and risk-adapted postoperative follow-up planning. External validation in larger, multi-center cohorts, assessment of imaging measurement reproducibility, and evaluation of real-world clinical impact are required before routine clinical implementation.

## Data Availability

The raw data supporting the conclusions of this article will be made available by the authors, without undue reservation.
