# Supplementary material for: A preoperative nomogram for predicting 2-year postoperative recurrence after percutaneous transforaminal endoscopic decompression in degenerative lumbar spinal stenosis
Source: Front Radiol. 2026 May 4;6:1821920. doi: 10.3389/fradi.2026.1821920 (PMC13180853; doi:10.3389/fradi.2026.1821920)
Supplement: Supplementary file 1 [file Image1.pdf]

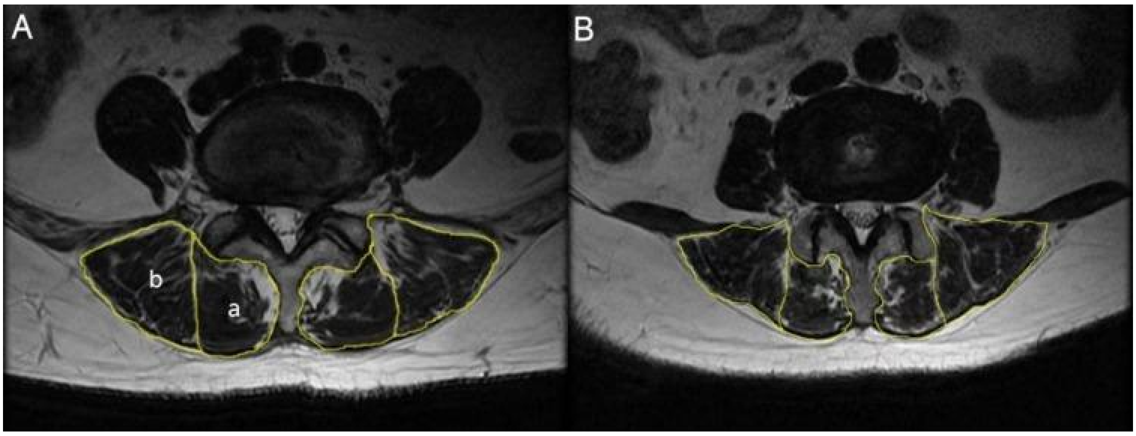

Supplementary Figure S1. Measurement of paraspinal muscle cross-sectional area and skeletal muscle index (SMI).

Yellow outlines indicate the manually delineated regions of interest (ROIs) on axial T2-weighted MRI at the mid–L3/4 disc level, encompassing the bilateral multifidus (a) and erector spinae (b) muscles. (A) Representative preoperative image from a non-recurrence patient (female, 63 years). (B) Representative preoperative image from a recurrence patient (female, 62 years). Images were imported into ImageJ (v1.8.0), and muscle borders were traced on each side to obtain cross-sectional areas. The paraspinal total cross-sectional area (TCSA) was calculated as the sum of the multifidus and erector spinae areas, with left and right sides summed to obtain a single patient-level bilateral TCSA. The skeletal muscle index (SMI) was computed as: SMI = TCSA (mm^2) / $height^2$ (m^2).
